# Medical toxicology: Need for recognition as a subspecialty

**DOI:** 10.4103/0253-7613.75655

**Published:** 2011-02

**Authors:** Aruna Dewan

**Affiliations:** Ex- Chief, Poison Information Centre, National Institute of Occupational Health (ICMR), Ahmedabad, India. E-mail: dewanaruna@yahoo.com

There are some disciplines in medical curriculum which are learnt grudgingly and ignored. Toxicology happens to be one of them. Most of it is taught as a part of Forensic Medicine, Pharmacology covers a little bit and Internal Medicine gives it the last spot. By the time an average medical student completes MBBS, toxicology means treatment of snake bite or organophosphate poisoning. However, in actual practice, toxicology is far more challenging. A simple definition of toxicology is the “Science of Poisons”. A more descriptive definition is “the study of the adverse effects of chemicals or physical agents on living organisms”. Toxicology is a broad discipline which combines the principles of Pharmacology, Biochemistry, Analytical Chemistry, Cell Biology and Environmental Sciences and many others. Its applications are wide and important.

Since the middle of the 20^th^ century, an ever increasing number of chemicals have been used for improving agricultural output and the manufacture of pharmaceutical and household products. The availability of these products has no doubt improved the quality of life but has also led to unintentional occupational and environmental exposures. Mass chemical poisonings have occurred due to contamination of food, use of spurious drugs and industrial accidents. India faced the worst industrial disaster due to methyl isocyanate in December 1984 at Bhopal. In the 1950s, poisoning was recognized as a leading cause of pediatric hospital admissions in the United States and this led to the creation of Poison Information Centers. Simultaneously, Clinical Toxicology developed as a subdiscipline of Medicine and the American Association of Poison Control Centers (AAPCC) was established in 1958. It was largely due to the efforts of AAPCC that Poison Prevention Packaging Act was introduced in 1972. This led to child resistant packaging and a marked reduction in childhood poisonings.

In the United States, Poison Centers (PCs) are staffed by a variety of medical professionals including medical and clinical toxicologists, registered nurses, pharmacists, chemists, hazardous material (HAZMAT) specialists, and epidemiologists. These centers are available at no charge to the caller, 24 hours a day, every day, and provide telephonic information. PCs respond to questions from the public and healthcare professionals. During 2007, 60 of the 61 US PCs uploaded the case data automatically into the National Poison Data System (NPDS). Over 4.2 million calls were captured by NPDS in 2007 of which 2,482,041 were human exposures and 83.2% exposures were unintentional. The agents most commonly involved were analgesics, cosmetics/personal care products, household cleaning substances, sedative/hypnotics and antipsychotic drugs. Pesticides were involved in only 3.9% cases. Specialized training in medical toxicology for physicians has been available since the 1970s. The American Academy of Clinical Toxicology established the American Board of Medical Toxicology (ABMT) in 1974. After much effort, American Board of Medical Specialties (ABMS) recognized medical toxicology as a subspecialty and the first ABMS-recognized examination in Medical Toxicology was offered in 1994.

In India, the exact number of toxic exposures is not known as there is no centralized reporting system. Studies published on hospital-based poisoning data from different parts of the country are the only source of information. Pesticides are the commonest agent involved and suicidal poisonings are much more common than accidental or occupational poisoning. Due to the easy access to highly toxic pesticides, which are used even in small farms, poisoning-related mortality is much higher and varies from 15 to 35%. Fatalities due to aluminum phosphide have been a great concern especially in northern parts of India. According to one estimate, deaths due to aluminum phosphide self-poisoning in India may be even more than those due to the Bhopal gas tragedy! Unlike organophosphates, there is no antidote for aluminum phosphide.

In recent times, there has also been a great concern about low dose chronic exposure to environmental chemicals such as persistent organic pollutants (POPs) and heavy metals like lead. Many of these chemicals are known to be carcinogens and endocrine disruptors. Neurobehavioral disorders in children are also being linked to *in-utero* exposures. The famous Hollywood movie “Erin Brockovich” is based on a true life story in which hexavalent chromium contamination of ground water caused a variety of illness in the residents of that area. The case was settled in 1996 for US $333 million, the largest settlement ever paid in a direct action lawsuit in US history. There are many examples of morbidity and mortality due to toxic substances. Chinese media reported in the beginning of September 2008 that some brands of infant formula were contaminated with melamine. More than 51,900 infants and young children in China were hospitalized for urinary problems, possibly renal tube blockage and kidney stones related to the consumption of melamine in powdered infant formula. Melamine is a chemical compound that has a number of industrial uses. The tainted milk powder has also been used in the manufacture of a number of other products. An outbreak of lead intoxication leading to a cluster of deaths in children in Senegal was reported in February 2009, and it was found to be due to informal and unsafe recycling of used lead-acid batteries.

The need for PCs has been recognized in India for the past two to three decades but not much has been achieved. The existing four PCs at New Delhi, Ahmedabad, Chennai and Cochin have started with the technical support of International Program on Chemical Safety (IPCS) and the World Health Organization (WHO) and personal efforts of a few individuals interested in medical toxicology. Unlike other branches of medicine, there are few takers for toxicology and treatment of poisoning is often arbitrary and follows obsolete practices. Facilities for toxicological analysis are almost non-existent even in tertiary care hospitals, though every healthcare facility handles poisoning cases. Many patients with severe poisoning die before they reach hospitals from far off areas. Nonavailability of treatment facilities, lack of qualified personnel and inherent toxicity of pesticides are the main reasons for this sorry state of affairs.

The subject of toxicology is heterogeneous and interdisciplinary. The core training requires teaching in prevention, monitoring, evaluation, diagnosis, and treatment of toxic exposures including intentional, unintentional, occupational and environmental exposures. Medical toxicology training also needs a close association with a poison control center. Unfortunately, we have neither adequate number of PCs nor a training program in medical toxicology in India.

Do we need another Bhopal to remind us of our negligence?

